# Deriving General Energy Predictors for Pnictogen Bonding

**DOI:** 10.1002/chem.71386

**Published:** 2026-07-16

**Authors:** Jan Langwald, Antonio Frontera

**Affiliations:** ^1^ Institute of Inorganic and Materials Chemistry University of Cologne Cologne Germany; ^2^ University of the Balearic Islands Institute of Organic Chemistry Carretera de Valldemossa km 7.5 Palma de Mallorca Spain

**Keywords:** density functional calculations, noncovalent interactions, pnictogen bonding, supramolecular chemistry

## Abstract

A comprehensive quantum chemical study is presented to derive energetic predictors for pnictogen (Pn) bonding interactions. We analyzed the interaction energies of the Lewis acids PnF_3_ and F_2_PnX, where Pn represents nitrogen through bismuth and X represents chlorine, bromine, or iodine, acting as σ‐hole donors with a diverse set of up to twenty‐one different nitrogen‐ and oxygen‐based Lewis bases. All complexes were investigated at the PBE0‐D3/def2‐TZVP level of theory. For every complex, a topological analysis according to Bader's quantum theory of atoms in molecules (QTAIM) was performed to correlate the interaction energies with the potential energy density values at the bond critical points. We established a linear correlation with reasonable R‐squared values for the coordination behavior of each pnictogen atom. Furthermore, the results demonstrate that, as the size, polarizability, and σ‐hole intensity of the pnictogen atom increase, the likelihood of forming additional secondary noncovalent interactions also rises. Consequently, a clear differentiation must be made between pure pnictogen bonding and a combination of pnictogen bonding with secondary interactions. This distinction is supported by the analysis of several published crystal structures. We propose general energy predictors for the determination of pure pnictogen bond energies.

## Graphical abstract

This study derives general energetic predictors for pure pnictogen bonds from nitrogen to bismuth based on the potential energy density at the bond critical point. The research highlights the increasing occurrence of secondary interactions for heavier elements.

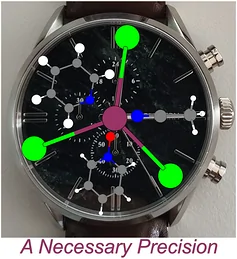

## 1 Introduction

Noncovalent interactions [1, 2, 3], more precisely σ‐hole and π‐hole interactions, describe the attractive forces between a region of positive electrostatic potential and a Lewis base [4, 5, 6]. This positive region is localized along the extension of a covalent bond for *σ*‐holes or perpendicular to a molecular framework for *π*‐holes. These interactions represent a rapidly growing field of research due to the realization that they exert significant structural stabilization effects across many systems [7, 8, 9, 10, 11, 12, 13, 14, 15, 16]. To date, the formation of noncovalent interactions has been extensively observed and studied for several main group elements and transition metals using quantum chemical calculations [17, 18, 19, 20, 21, 22, 23, 24], solid‐state NMR [25, 26, 27], and XPS techniques [27, 28].

Pnictogen bonding is the noncovalent interaction between a suitable pnictogen‐based *σ*‐hole donor, such as a PnX_3_ compound, and a Lewis base [29, 30]. The potential applications of pnictogen bonding have been widely investigated in recent years, spanning supramolecular [31, 32, 33, 34, 35, 36] and coordination chemistry [37, 38, 39, 40], anion recognition and transport [41, 42, 43, 44, 45, 46], organic synthesis and catalysis [47, 48, 49, 50, 51], as well as biochemical applications like the design of artificial enzymes [52]. However, a quantitative evaluation of the proposed pnictogen bonds is usually lacking, and topological analyses are performed only in rare cases. Due to the comparable or larger atomic size relative to group sixteen and seventeen elements, combined with the availability of three *σ*‐holes in trivalent pnictogen derivatives, the likelihood of encountering additional secondary interactions alongside pure pnictogen bonding is starkly increased compared to chalcogen and halogen bonding [53].

The groundbreaking work by *Espinosa* et al. [54] on suitable energy predictors for hydrogen bonding served as the foundation for this methodology (see Figure 1). Subsequently, similar predictors have been successfully derived for halogen and chalcogen bonding [55, 56]. The present study aims to extend these reports by deriving suitable energy predictors for pnictogen bonding, which may facilitate the quantification of these noncovalent interactions in future studies. However, since the existence of an additional *σ*‐hole located opposite to the pnictogen lone pair has been demonstrated by previous research [57], particularly when the atom is coordinated to a metal, a clear differentiation must be made between unconventional pnictogen bonds involving this specific *σ*‐hole and classical ones involving any of the remaining three *σ*‐holes.

**FIGURE 1:**
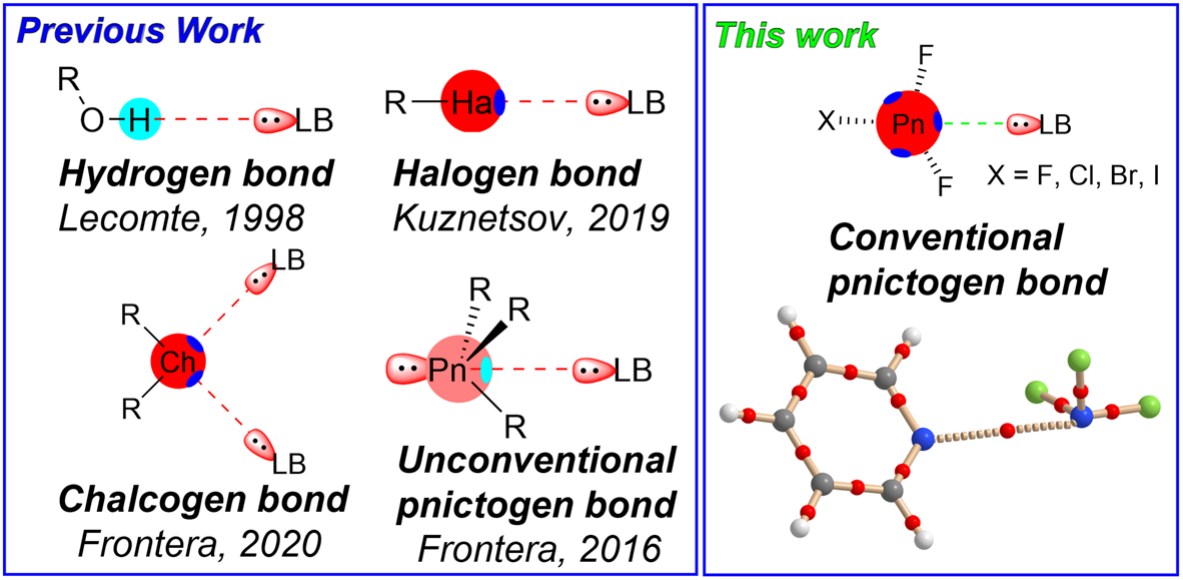
Overview of the noncovalent interactions for which energy predictors have been described (except for unconventional pnictogen bonding).

## 2 Results and Discussion

To get as close as possible to the evaluation of pure pnictogen bonds, we predominantly utilized PnF_3_ compounds, where Pn ranges from nitrogen to bismuth, as *σ*‐hole donors because they exhibit the strongest positive electrostatic potentials and negligible steric influence. We also included F_2_PnX derivatives, where X represents chlorine, bromine, or iodine, to assess the influence of a decreasing *σ*‐hole intensity. In these unsymmetrical cases, the studied interactions always involve the coordination of the Lewis base toward the *σ*‐hole located opposite to the specific Pn─X bond. The respective *σ*‐hole strengths can be evaluated from the molecular electrostatic potential maps. For the collection of Lewis acids considered in this work, the maximum electrostatic potential values are summarized in Table 1.

**TABLE 1:** Molecular electrostatic potential (in kcal/mol) for the *σ*‐holes used for the complex formation of the studied Lewis acids. For the F_2_PnX species (X = Cl, Br, and I), the MEP value on the extension of the X─Pn bond is given in kcal/mol.

Pn	PnF_3_	PnF_2_Cl	PnF_2_Br	PnF_2_I
N	15.6	9.9	9.6	5.7
P	26.2	26.3	27.1	26.2
As	38	34.3	33.4	30.6
Sb	46.2	42	40.8	38
Bi	55.4	51.5	49.9	46.4

As expected, the intensity of the *σ*‐hole increases with the increasing atomic size of the pnictogen element and decreases with the decreasing electronegativity of the halogen substituent. Notably, the difference between the PnF_3_ and F_2_PnX species is significant for nitrogen, whereas it is only moderate for arsenic, antimony, and bismuth, and it virtually vanishes for phosphorus.

As Lewis bases, we investigated a broad portfolio of nitrogen‐ and oxygen‐containing donors derived from nitriles, pyridine, and pyridine‐N‐oxide derivatives. An overview of the specific Lewis acids and bases used in the calculations is summarized in Figure 2. The full datasets are provided in the Supporting Information showing the typical behavior of *σ*‐hole interactions. That is, stronger interaction energies are observed to the heavier pnictogen atoms (see Tables S1 to S5). For every complex, geometry optimization without symmetry constraints was initially performed at the PBE0‐D3/def2‐TZVP level of theory [58, 59, 60]. In some cases, the optimizations were repeated using the C_S_ symmetry constraint (see below) to avoid the formation of secondary interactions, that was effective in a few examples. Subsequently, a topological analysis of the electron density was carried out using a QTAIM analysis [61].

**FIGURE 2:**
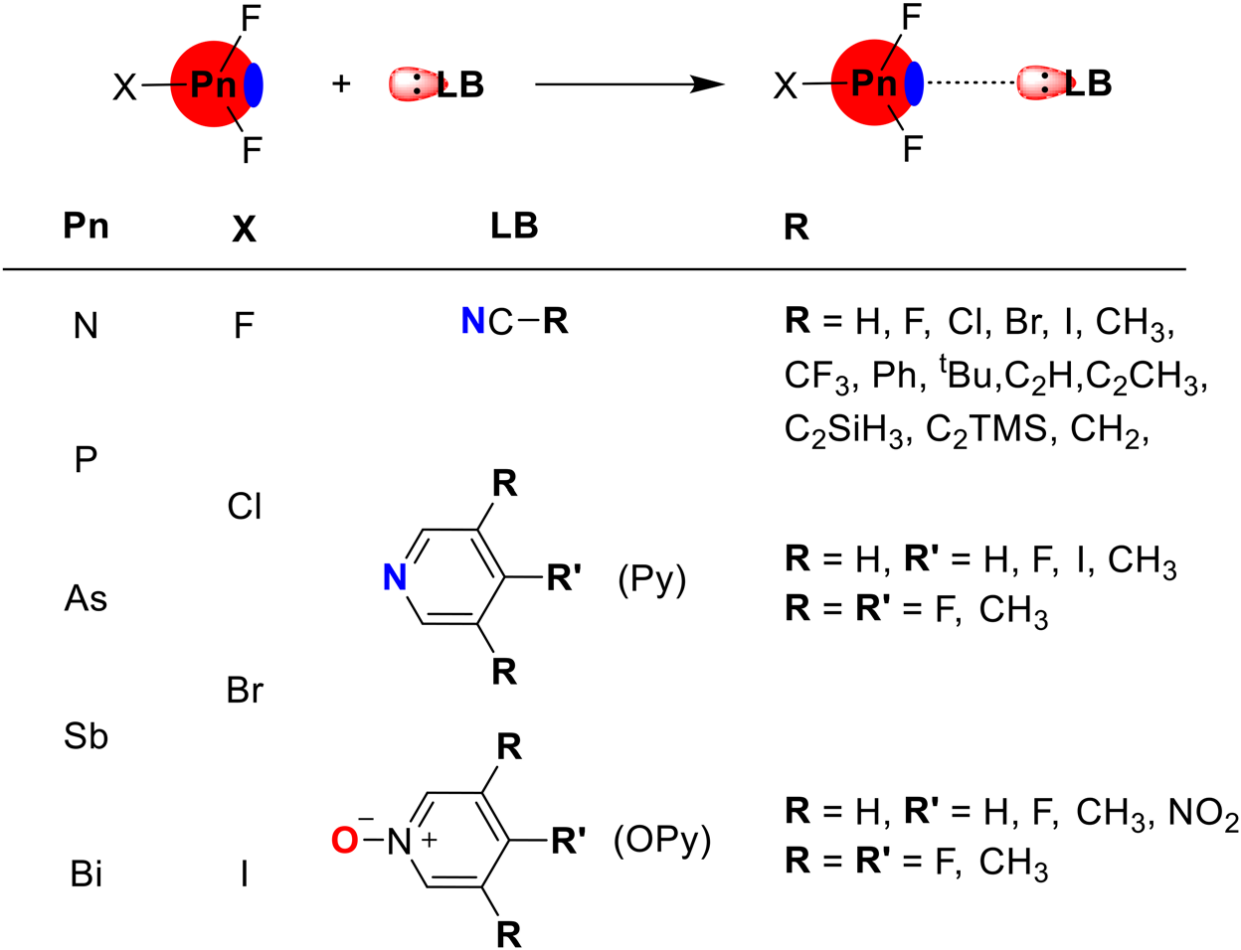
Overview of the Pnictogen compounds and Lewis bases used for the presented study. In the following, abbreviations based on the substituents will be used, for example F_3_N∙∙∙Py4F or F_2_ClSb∙∙∙OPy345Me.

We investigated whether a mathematical correlation between the interaction energies and several QTAIM parameters at the bond critical point (BCP) connecting the pnictogen to the electron‐rich atom could be established. Among these parameters, we selected the potential energy density because it provided the best correlations for the entire series of pnictogen atoms. For the NX_3_ and PX_3_ species, the correlation plots of the interaction energies as a function of the potential energy density are shown in Figure 3.

**FIGURE 3:**
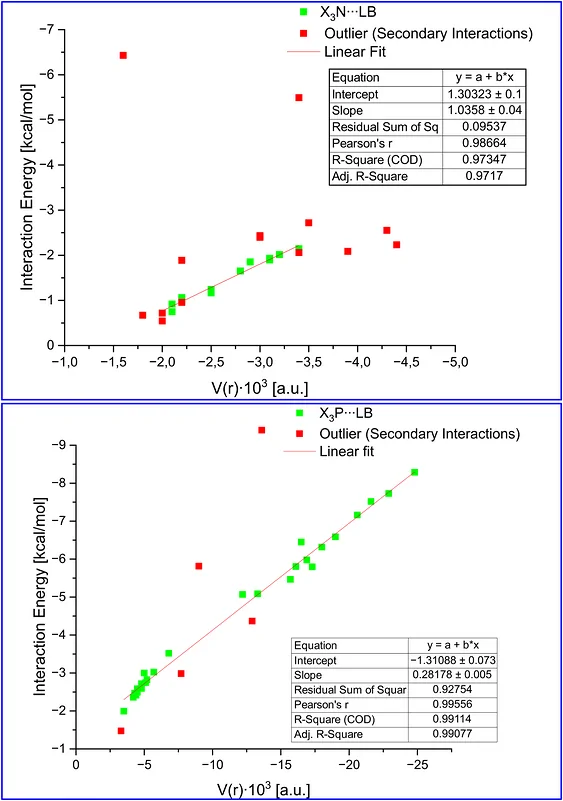
Plots of the interaction energies for the X_3_Pn∙∙∙LB (Pn = N [top] and P[bottom] complexes as a function of *V*(*r*). The green and red colors indicate the formation of ‘pure’ pnictogen bonds and the development of secondary interactions (e.g. H–bonding), respectively. The red line represents a linear fit for the pure pnictogen bonding datapoints with the respective mathematical details shown in the inset table.

For nitrogen, as the smallest pnictogen, the role as a *σ*‐hole donor is uncommon because it usually acts as a Lewis base due to the strong nucleophilicity of its lone pair [62]. However, the respective *σ*‐holes in the extension of the covalent N─X bonds still show significant electropositive potentials [57], as demonstrated by the MEP calculations (see Table 1). Thus, pnictogen bonding between NF_3_ and suitable Lewis bases can occur and does indeed correlate with the stabilization energy of the formed complexes, as shown in Figure 3 (top).

Due to the small size of the nitrogen atom and the relatively short N─F bond lengths, the Lewis bases come comparably close to the NX_3_ unit upon complexation. We noticed that this spatial proximity fosters the formation of secondary interactions, such as hydrogen bonding with hydrogen‐bearing Lewis bases like pyridine, or fluorine‐carbon interactions in the case of perpendicular aromatic *π*‐systems like pyridine‐N‐oxide. In fact, we observed the formation of secondary interactions for all investigated pnictogen compounds, whereby it is the least prominent for phosphorus and increases with the increasing size, polarizability, and positive potential of the *σ*‐hole of the pnictogen atom.

At first, we observed the formation of additional hydrogen bonding between the fluorine atoms of the PnF_3_ unit and suitable hydrogen bond donors, such as pyridine. In several of these cases, we were able to isolate the pure pnictogen bonding interaction by imposing C_S_ symmetry during the optimization process, as illustrated in Figure 4 (top).

**FIGURE 4:**
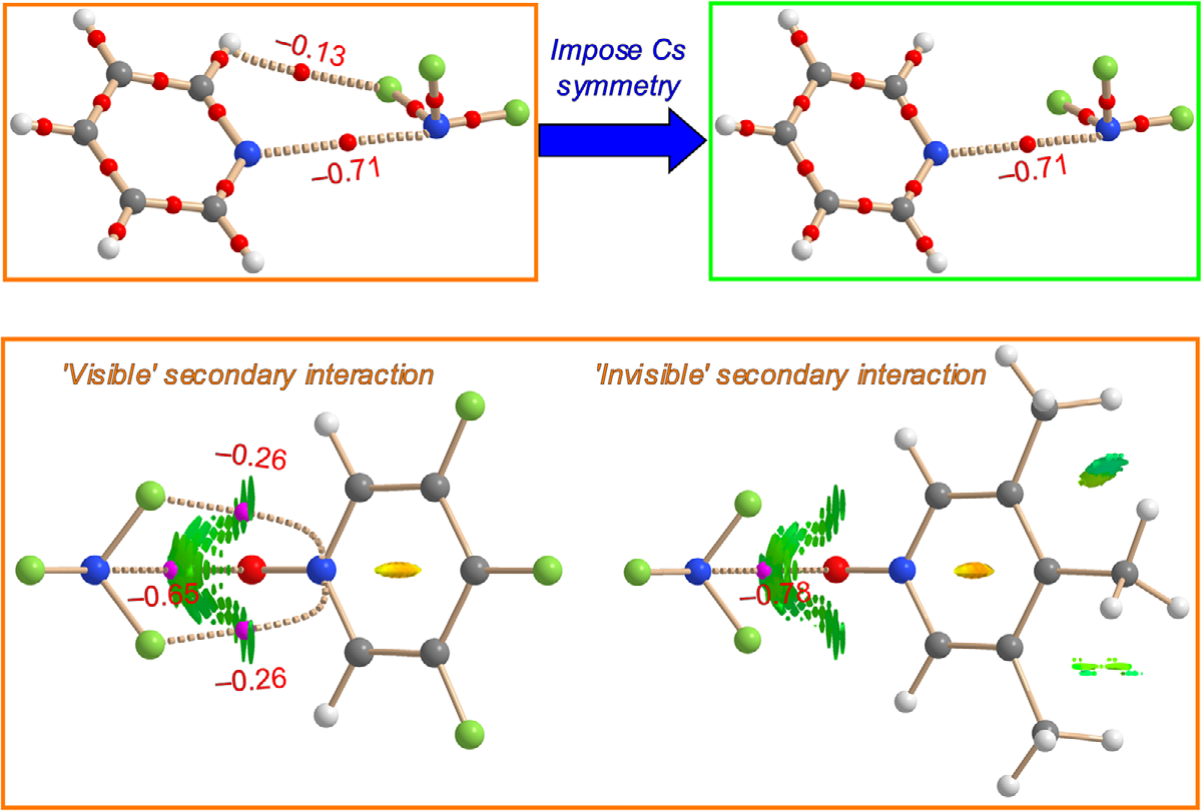
Coordination and topological analysis of the present NCIs in F_3_N∙∙∙py before (top left) and after (top right) imposing Cs symmetry for the calculation. For larger systems with suitable donor/acceptor functions, the formation of secondary interactions cannot be resolved by imposing a symmetry (bottom left). In some cases, although the presence of secondary interactions is not revealed by the topological analysis, their influence can be mapped by looking at the RDG plots (bottom right). Isosurfaces range from 0.04 (red) to –0.04 a.u. (blue). The values of *V*(*r*) × 10^3^ at the bond critical points are indicated.

For several systems that are classified as outliers, the QTAIM topological analysis did not show the presence of additional bond critical points and bond paths interconnecting the *σ*‐hole donor and acceptor molecules. In these cases, only the NCIplot analysis, via the respective reduced density gradient (RDG) isosurfaces, was able to disclose weak interactions that coincide with the position of BCPs in related systems, as shown in Figure 4 (bottom). These cases where the secondary interactions are very weak correspond to the red outliers that visually lie on the regression line, illustrating that there is a very thin line between the development of pure pnictogen bonds and the additional influence from secondary interactions. Some of these outliers from the nitrogen systems would in general fit the overall linear correlation, as shown in Figure 3 (top); however, to maintain strict methodological consistency, we have excluded them from the final mathematical regression plot.

When moving toward the heavier pnictogens arsenic and antimony, as shown in Figure 5, the differentiation between the pure interactions and those where secondary interactions are present becomes progressively easier due to significant differences in the interaction energies and the range of derived *V*(*r*) values. This is in line with what would be expected due to the increasing size and strength of the available *σ*‐holes at the pnictogen atom.

**FIGURE 5:**
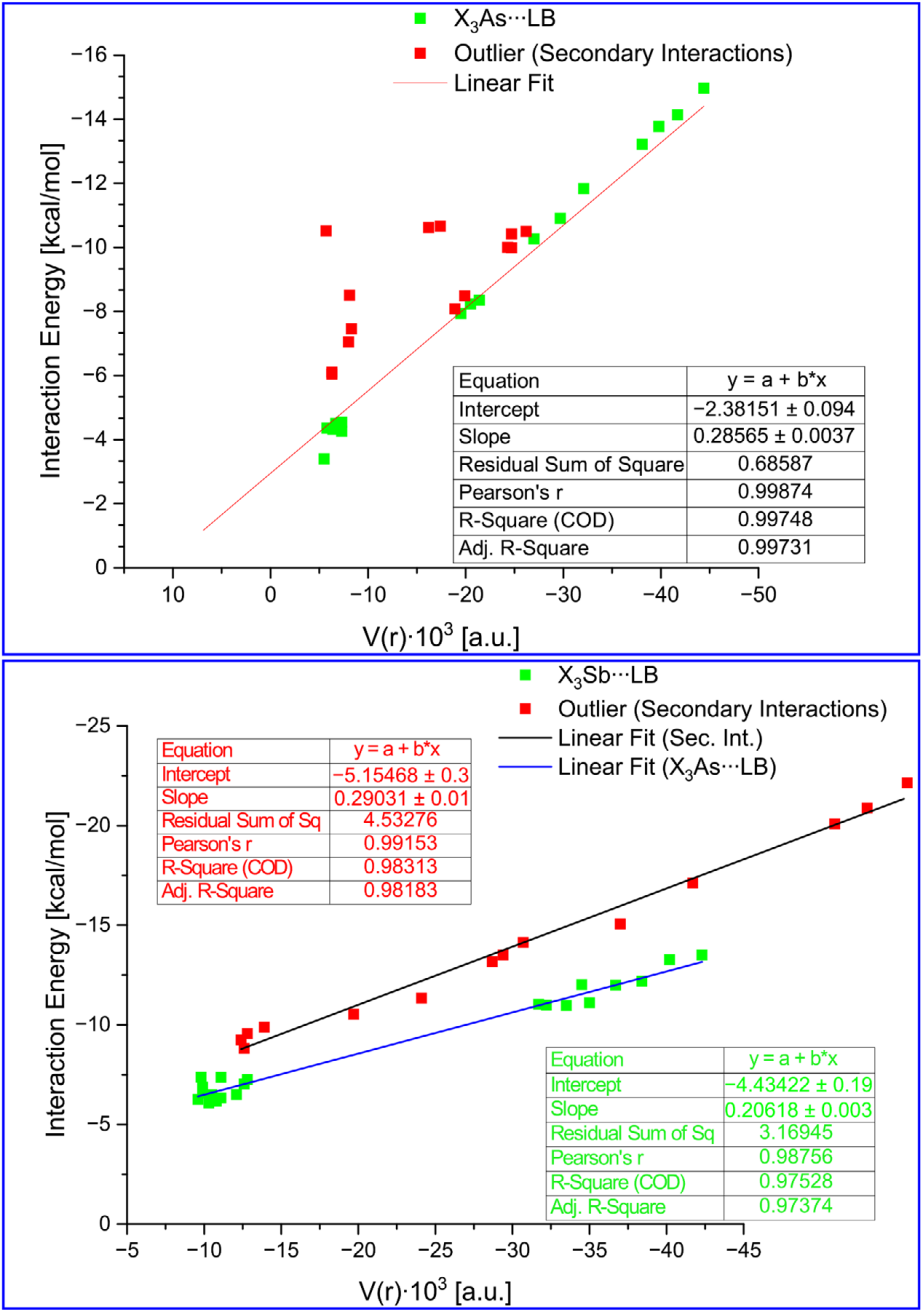
Plots of the interaction energies for the X_3_Pn∙∙∙LB (Pn = As [top] and Sb [bottom] complexes as a function of *V*(*r*). The green and red colors indicate the formation of ‘pure’ pnictogen bonds and the development of secondary interactions (e.g. H–bonding), respectively. The red and black lines represent linear fits for the pure pnictogen bonding and secondary interaction assisted pnictogen bonding datapoints, with the respective mathematical details shown in the inset tables.

Notably, for antimony, all outliers, both with and without imposed C_S_ symmetry, show secondary interactions. These primarily consist of C···F or H···F interactions between the remaining fluorine ligands at the pnictogen and the carbon atoms of the pyridine ring of pyridine and pyridine‐N‐oxide, as well as with the protons of phenyl groups in benzonitrile or hydrogen bonds of methyl groups in tert‐butyl nitrile.

The energy values derived for these systems also follow a linear correlation, as shown in Figure 5 (bottom). The slope of the respective linear fit is steeper compared to the pure pnictogen bonds. This is due to the extensive increase in interaction energy upon the addition of several different noncovalent interactions, which all contribute to the overall stabilization of the complex. We also observed that the gaps in the middle sections of the plots for antimony and bismuth could not be resolved.

Although the pnictogen bonding strength can be correlated quite well for nitrogen through antimony, the increasing effect of secondary interactions and the resulting deviation from the models derived for pure pnictogen‐based noncovalent interactions are evident from the increasing residual sum of squares for the linear fits. This trend culminates in the results for bismuth, where we could only use a small selection of Lewis bases for the complexation (See Table S5), as shown in Figure 6, due to the high tendency to establish secondary interactions. The larger scatter observed for the bismuth correlations compared to the lighter pnictogens arises from a combination of both factors: the exceptionally high polarizability of bismuth and the increased occurrence of secondary interactions. On one hand, the extreme polarizability of Bi leads to a more diffuse electron density distribution at the *σ*‐hole, making the *V*(*r*) at the BCP a less precise descriptor of the interaction strength than for lighter pnictogens. On the other hand, even for complexes classified as ‘pure’ pnictogen bonds, residual long‐range secondary interactions, too weak to produce detectable BCPs yet energetically nonnegligible, contribute to additional scatter. Both effects are inherently linked: the large, polarizable Bi atom not only perturbs the electron density more strongly upon complexation but also provides a larger van der Waals surface that facilitates the onset of such secondary contacts.

**FIGURE 6:**
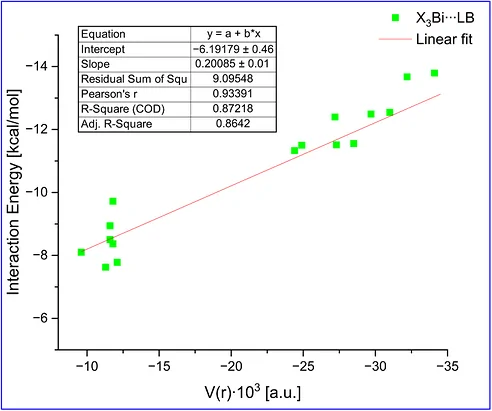
Plots of the interaction energies for the X_3_Bi∙∙∙LB complexes as a function of *V*(*r*). The green color indicates the formation of ‘pure’ pnictogen bonds. The red line represents a linear fit for the pure pnictogen bonding datapoints with the respective mathematical details shown in the inset table.

The variation observed in the *b* values of the equations in the regression plots (1.036 for N, 0.282 for P, 0.286 for As, 0.206 for Sb, and 0.201 for Bi), which represent the slope of the correlation between the interaction energy and the *V*(*r*) at the BCP, provides valuable insights into the changing physical nature of the pnictogen bonding interaction as group 15 is descended. This stepwise decrease and the clustering of the slopes reflect a clear transition in the underlying forces governing these noncovalent interactions, moving from electrostatic dominance to increased polarizability and orbital interaction.

For the lightest congener, nitrogen, the slope is exceptionally large. This behavior is rationalized by the high electronegativity and small covalent radius of nitrogen, which results in low polarizability. Nitrogen rarely acts as a *σ*‐hole donor, and when it does, the interaction is weak and almost purely electrostatic, requiring a different sensitivity in the energy predictor compared to its heavier counterparts.

When moving to phosphorus and arsenic, the slope values drop significantly and become similar, with *b* values around 0.28. In these intermediate elements, the *σ*‐hole becomes much more pronounced, and the interaction begins to receive notable contributions from dispersion and charge transfer effects. The close agreement between the slopes of phosphorus and arsenic indicates that within this region of the periodic table, the balance between electrostatic and dispersion or covalent components remains highly uniform across the series of Lewis bases.

A further decrease in the slope is observed for the heaviest elements, antimony, and bismuth, where the *b* values settle around 0.20. As the pnictogen atom becomes larger, the polarizability increases drastically, making charge transfer, and orbital mixing much more prominent features of the pnictogen bond. For bismuth, this effect is further enhanced by relativistic contributions, which cause a contraction of the core *s* orbitals and a subsequent expansion and shielding of the valence *p* orbitals. This increase in electron diffuseness and polarizability allows a smaller *V*(*r*) at the BCP to accommodate a relatively stronger interaction energy.

Consequently, the progression of the slope values from nitrogen down to bismuth physically captures the gradual shift from a highly localized, weakly electrostatic interaction to a more diffuse, polarizable, and partially covalent noncovalent bond.

Although a good correlation between the calculated interaction energies and the *V*(*r*) values is generally observed, the accuracy of the derived functions decreases with the increasing atomic size of the pnictogen, most likely due to the existence of long‐range interactions and the poorer directionality of the contact due to the large size of the *σ*‐hole.

In many reported crystal structures, as well as in the few quantum chemical investigations of crystal structures containing pnictogen bonds, it is evident from the chemical environment of the PnX_3_ species that additional noncovalent interactions are present [63, 64, 65, 66, 67]. Yet, in nearly all cases, only the pnictogen bond is discussed, without further elucidation of the secondary interactions. Furthermore, even the heavier pnictogen atoms still possess a lone pair, which in electron‐rich compounds like trimethylbismuthine act as the Lewis base when combined with suitable *σ*‐hole donors, such as iodopentafluorobenzene [68].

To visualize these differences, we selected three published crystal structures from previous reports: the dimeric cocrystal of 4,4‐dipyridyl dioxide and antimony trifluoride (CCDC 1956889) [69] featuring a rigid organic backbone, the flexible crown ether complex of 18‐crown‐6 and antimony trifluoride (CCDC 1236624) [70], and the dimeric alkoxide‐caged arsenic compound (CCDC 1940303) [34]. We isolated the respective molecular assemblies using the software Mercury [71] using the X‐ray geometries and performed single‐point energy calculations at the PBE0‐D3/def2‐TZVP level of theory, followed by a topological analysis using QTAIM. The results are shown in Figure 7. For the 4,4‐dipyridyl dioxide antimony trifluoride complex, several secondary fluorine‐hydrogen, oxygen‐carbon, and carbon‐carbon interactions are present characterized by the corresponding BCP and bond paths. This is in line with the results previously observed for our model systems combining heavier pnictogen

**FIGURE 7:**
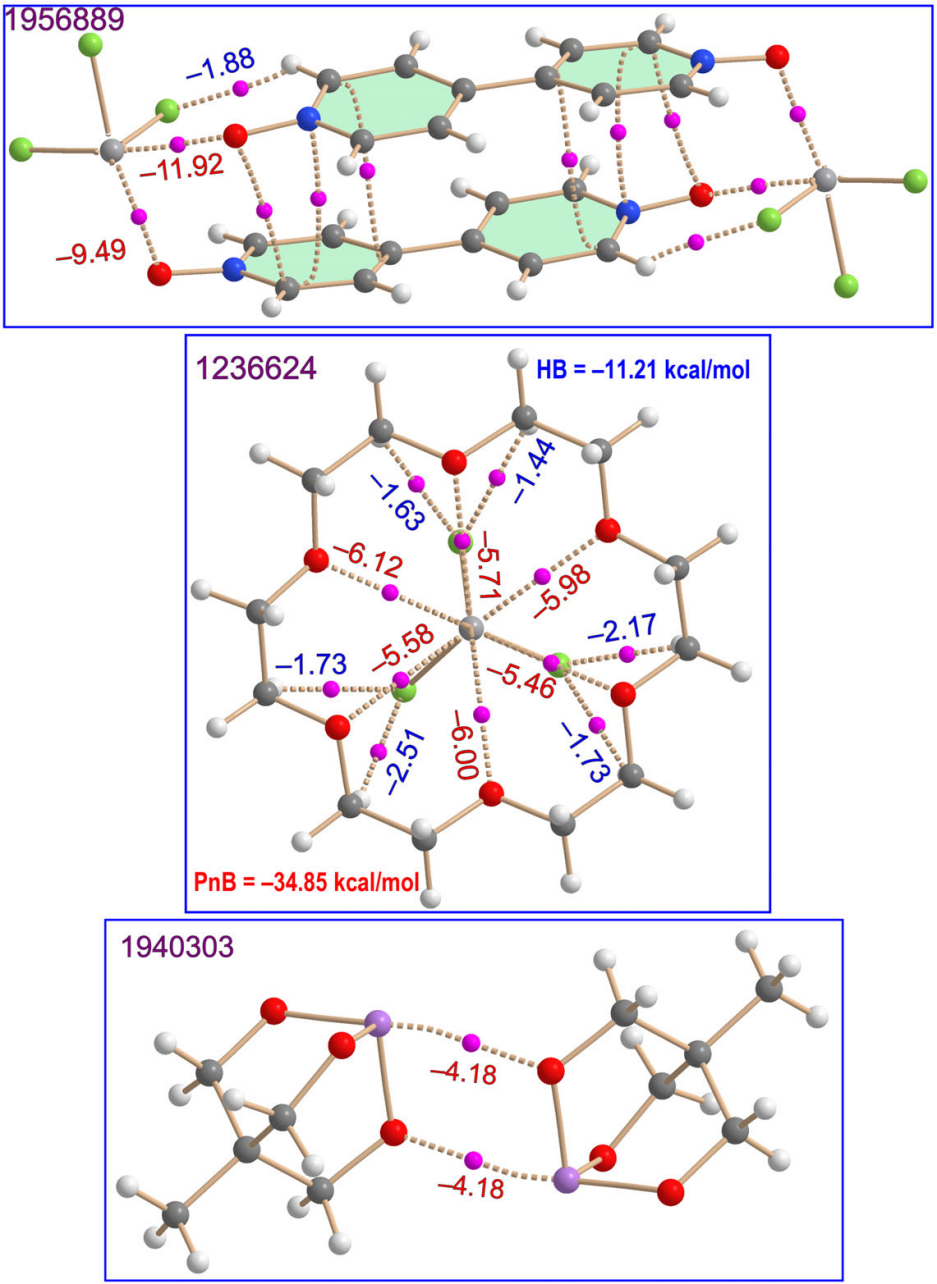
Overview of the noncovalent interactions present in selected reported solid‐state structures using QTAIM analysis (BCPs in fuchsia and bond paths as dashed bonds), calculated at the PBE0‐D3/def2‐TZVP level of theory (CCDC numbers are given at the top left corner of every picture). The pnictogen bonding and secondary interaction energies calculated from energy predictors are colored red and blue, respectively and are given in [kcal/mol].

*σ*‐hole donors with aromatic Lewis bases. The most dominant interaction energies stem from the oxygen‐antimony pnictogen bonds (–9.49 kcal/mol and –11.92 kcal/mol) deduced using the equation derived by the regression of Figure 5. The contribution from the *π*‐stacking was estimated by eliminating the AsF_3_ molecules and computing the dimerization energy of the resulting dimer, which is –6.42 kcal/mol. Finally, the sum of the hydrogen bonds estimated using Espinosa's equation accounts for –3.77 kcal/mol, revealing a nonnegligible contribution of the C─H···F contacts. The total stabilization energy of the assembly calculated using the supramolecular approach is –49.17 kcal/mol. If we subtract the contribution of the *π*‐stacking and H‐bonds, the remaining stabilization energy is –38.99 kcal/mol. This value correlates reasonably well with the sum of the pnictogen bond energies calculated using our predictor (–42.83 kcal/mol) thus providing reliability to the equation.

A similar situation is observed for the crown ether complex with SbF_3_, which is one of several crystallographically characterized oxo‐, aza‐, or thia‐crown ether complexes with PnF_3_ species [66, 69, 71, 72, 73, 74]. In this case, the energetic contributions from pure pnictogen bonding (–34.85 kcal/mol) are significantly larger than the sum of the fluorine‐hydrogen interactions (–11.21 kcal/mol), evidencing that the pnictogen bonds are dominant. In this example, the total interaction energy estimated by the combined pnictogen bond and hydrogen bond predictors is –46.06 kcal/mol. This energy is larger than the value obtained by the supramolecular approach, where the interaction energy is computed as the difference between the complex and the separated monomers, which yields –38.10 kcal/mol. This discrepancy is most likely due to the bifurcated nature of the pnictogen bonds. That is, each *σ*‐hole at the SbF_3_ unit simultaneously establishes two pnictogen bonds with two oxygen atoms of the crown ether. Because the energy predictor was not trained on bifurcated pnictogen bonds, the individual pnictogen bonding contributions are most likely overestimated, thus explaining the difference of approximately 8 kcal/mol between the computed binding energy and the value estimated by summing the predicted hydrogen bond and pnictogen bond energies. Therefore, the proposed energy predictors should be applied with caution in systems featuring bifurcated pnictogen bonds or highly crowded supramolecular cavities where cooperative multi‐center effects are operative, as well as for bismuth derivatives where the high polarizability of the Bi atom reduces the precision of the linear correlation.

In the case of the dimeric arsenic compound shown in Figure 7, bottom, pure pnictogen bonding between adjacent oxygen and arsenic atoms is observed, with a lack of secondary interactions. The calculated interaction energy for each contact using the supramolecular approach is –4.50 kcal/mol and the value estimated with our energy predictor (–4.18 kcal/mol) are in excellent agreement, supporting that our model serves as a reliable energy predictor for pure pnictogen bonds.

## 3 Conclusion

The interaction energies of the Lewis acids PnF_3_ and F_2_PnX, where Pn ranges from nitrogen to bismuth and X represents chlorine, bromine, or iodine, acting as *σ*‐hole donors with up to twenty‐one different nitrogen‐ and oxygen‐based Lewis bases were calculated at the PBE0‐D3/def2‐TZVP level of theory. This was followed by a topological QTAIM analysis. Very good linear correlations between the interaction energies and the potential energy density values at the bond critical points were established for nitrogen through antimony, while the correlation quality slightly decreased for the bismuth derivatives. With the increasing atomic size of the pnictogen, the formation of additional secondary noncovalent interactions increases accordingly, which necessitates a clear differentiation between pure pnictogen bonding and these secondary contributions. By analyzing different examples from reported crystal structures, we demonstrated that an additional influence of secondary interactions alongside pnictogen bonding is frequently present, but the derived interaction energies consistently correlate with our model systems. A current limitation of the derived predictors lies in their application to cooperative or multi‐center environments; specifically, the models may overestimate individual contributions in bifurcated pnictogen bonds, a factor that should be taken into account when analyzing highly crowded supramolecular cavities. Furthermore, the predictive accuracy is somewhat reduced for bismuth derivatives; due to the high polarizability of the heavy Bi atom and the resulting clustering of data points, the derived linear fit exhibits a higher degree of uncertainty compared to the lighter pnictogens.

From these analyses, we propose general energy predictors for the calculation of pure pnictogen bond energies based on the following equations (Equations (1), (2), (3), (4), (5)):

(1) $$E_{int}=1.30+1.04\times V\left(r\right)\mathrm{for}\;N$$

(2) $$E_{int}=-1.31+0.28\times V\left(r\right)\mathrm{for}\;P$$

(3) $$E_{int}=-2.38+0.28\times V\left(r\right)\mathrm{for}\;\mathrm{As}$$

(4) $$E_{int}=-4.34+0.21\times V\left(r\right)\mathrm{for}\;\mathrm{Sb}$$

(5) $$E_{int}=-6.20+0.20\times V\left(r\right)\mathrm{for}\;\mathrm{Bi}$$

This predictive method can be highly useful for researchers working in supramolecular chemistry and crystal engineering, as it provides a practical tool to isolate and quantify the specific contribution of pnictogen bonds within complex supramolecular assemblies. Through these investigations, we highlight the importance of a precise identification of every individual noncovalent interaction and its specific contribution to the total interaction energy. The more accurately all active noncovalent interactions are differentiated and classified, the easier it becomes to establish general guidelines for molecular architecture, ultimately aiding the rational design of new functional materials and catalysts based on these *σ*‐hole interactions.

## 4 Theoretical Methods

The quantum chemical computations were performed using the Gaussian 16 software package (Revision C.01) [75]. Full geometry optimizations were performed without symmetry constraints unless otherwise noted. For the X‐ray structures retrieved from the CSD, crystal coordinates from X‐ray diffraction were used without further geometry optimization to preserve the experimental supramolecular configurations. The electronic structure was resolved at the PBE0‐D3/def2‐TZVP level of theory [58, 59, 60]. Topological analysis of the electron density was conducted according to Bader's quantum theory of atoms in molecules (QTAIM) [61]. Wavefunction files from Gaussian were analyzed with the AIMAll software (version 19.10.12) [76]. Bond critical points (BCPs), bond paths, and several QTAIM parameters at these BCPs were determined for all relevant contacts. The bond interaction energies for the experimental geometries and the complexes with several contacts were obtained through the energy descriptors calculated herein (for pnictogen bonds) as well as the predictor of Espinosa et al. (*E*_int_ = 0.5 *V*(*r*) for hydrogen bonds), based on the potential energy density (*V*) found in the corresponding BCP [54]. All output files from the calculations are provided as supplementary materials.

## Conflicts of Interest

The authors declare no conflicts of interest.

## Supporting information




**Supporting File 1**: chem71386‐sup‐0001‐SuppMat.docx.

**Supporting File 2**: chem71386‐sup‐0002‐DataFiles.zip.

## Data Availability

All the data is provided in the Supporting Information (output files from the optimizations).
