# Supplementary material for: Deriving General Energy Predictors for Pnictogen Bonding
Source: Chemistry. 2026 Jul 16;32(39):e71386. doi: 10.1002/chem.71386 (PMC13715474; doi:10.1002/chem.71386)
Supplement: Supplementary file 1 [file CHEM-32-e71386-s001.docx]

**Deriving General Energy Predictors for Pnictogen Bonding.**

Jan Langwald,*^ab^ and Antonio Frontera ^*b^

*^a^ Institute of Inorganic and Materials Chemistry, University of Cologne, Greinstr. 6, 50939 Cologne, Germany.
E-mail: jan.langwald@uni-koeln.de
^b^ Universitat de les Illes Balears, Crta de Valldemossa km 7.5, 07122 Palma de Mallorca, Baleares, Spain.
E-mail: toni.frontera@uib.es*

**Table of Contents**

[1 – Nitrogen	2](#_Toc230004398)

[2 – Phosphorous	3](#_Toc230004399)

[3 – Arsenic	4](#_Toc230004400)

[4 – Antimony	6](#_Toc230004401)

[5 – Bismuth	7](#_Toc230004402)

# 1 – Nitrogen

**Table S 1:** Summary of the used complexes for the pnictogen bond calculations. The electronic energy of the complex, the energy difference ΔE (complex – starting materials), the distance Pn···LB and the values for V(r) and G(r) are given. Values for calculation that resulted in ‘pure’ pnictogen bonding are colored in green, whereas the values that are colored in orange indicate the presence of secondary interactions (e.g. H–bonding, C···F interactions, …).

| **No** | **Complex** | **Electronic Energy**  **[Hartree]** | **Δ E**  **[Hartree]** | **Δ E**  **[kcal/mol]** | **d (N···LB)**  **[**[**Å**](https://en.wikipedia.org/wiki/%C3%85)**]** | **V(r)**  **[a.u.]** | **Ρ**  **[a.u.]** | **∇ρ^2^**  **[a.u.]** | **G(r)**  **[a.u.]** |
| --- | --- | --- | --- | --- | --- | --- | --- | --- | --- |
| 1 | F3N···NCH | -447.252638 | -0.001611 | -1.011 | 3.285 | -0.0022 | 0.00408 | 0.018148 | 0.0034 |
| 2 | F3N···Py(2) | -601.987033 | -0.003022 | -1.896 | 3.215 | -0.0031 | 0.005546 | 0.02190 | 0.0043 |
| 3 | F3N···OMe2 | -508.813837 | -0.003417 | -2.144 | 3.119 | -0.0034 | 0.005508 | 0.024474 | 0.0047 |
| 4 | F2NCl···Py (Cl hole) | -962.271656 | -0.002629 | -1.650 | 3.269 | -0.0028 | 0.005004 | 0.020083 | 0.0039 |
| 5 | F2NBr···NCH (Br hole) | -2921.33747 | -0.001195 | -0.750 | 3.315 | -0.0021 | 0.003861 | 0.017423 | 0.0032 |
| 6 | F3N···NCCH3 | -486.549418 | -0.001855 | -1.164 | 3.250 | -0.0025 | 0.004365 | 0.019507 | 0.0037 |
| 7 | F3N···NCCF3 | -784.118482 | -0.001464 | -0.919 | 3.308 | -0.0021 | 0.003712 | 0.017151 | 0.0032 |
| 8 | F3N···NCtBu | -604.391088 | -0.001977 | -1.241 | 3.245 | -0.0025 | 0.004421 | 0.019695 | 0.0037 |
| 9 | F3N···NCPh | -678.128312 | -0.001883 | -1.182 | 3.246 | -0.0025 | 0.004370 | 0.019626 | 0.0037 |
| 10 | F3N···Py4Me | -641.272398 | -0.003078 | -1.931 | 3.224 | -0.0031 | 0.005432 | 0.021640 | 0.0042 |
| 11 | F3N···Py4I | -899.110346 | -0.002953 | -1.853 | 3.246 | -0.0029 | 0.005133 | 0.020809 | 0.0041 |
| 12 | F3N···Py345Me | -719.839748 | -0.003211 | -2.015 | 3.213 | -0.0032 | 0.005572 | 0.022074 | 0.0043 |
| 13 | F3N···NCF | -546.422068 | -0.001584 | -0.994 | 3.283 | -0.0022 | 0.003911 | 0.017901 | 0.0033 |
| 14 | F3N···NCCl | -906.712523 | -0.00164 | -1.029 | 3.282 | -0.0022 | 0.003970 | 0.018056 | 0.0034 |
| 15 | F3N···NCBr | -3020.50776 | -0.00165 | -1.035 | 3.283 | -0.0022 | -0.003970 | 0.018034 | 0.0034 |
| 16 | F3N···NCI | -744.374163 | -0.001691 | -1.061 | 3.281 | -0.0022 | 0.004007 | 0.018136 | 0.0034 |
|  |  |  |  |  |  |  |  |  |  |
| 17 | F2NCl···NCH (Cl hole) | -807.537185 | -0.001142 | -0.717 | 3.331 | -0.002 | 0.003931 | 0.017912 | 0.0031 |
| 18 | F3N···THF | -586.178464 | -0.008751 | -5.491 | 3.086 | -0.0034 | 0.005405 | 0.025209 | 0.0049 |
| 19 | F3N···NMe3 | -528.232472 | -0.004067 | -2.552 | 3.126 | -0.0043 | 0.007331 | 0.026833 | 0.0055 |
| 20 | F3N···Py | -601.987021 | -0.00301 | -1.889 | 3.218 | -0.0022 | 0.005518 | 0.021927 | 0.0032 |
| 21 | F3N···N(CF3)3 | -1421.03301 | -0.010249 | -6.431 | 3.461 | -0.0016 | 0.003234 | 0.013314 | 0.0024 |
| 22 | H3N···NHC | -149.862109 | -0.003556 | -2.231 | 2.423 | -0.0044 | 0.009216 | 0.033478 | 0.0064 |
| 23 | NCl3···NHC | -1528.12376 | -0.001071 | -0.672 | 3.411 | 0.003461 | 0.015731 | 0.0029 | 0.0033 |
| 24 | F2NCl···NHC | -807.537569 | -0.001526 | -0.958 | 3.309 | 0.003931 | 0.017912 | 0.0033 | 0.0033 |
| 25 | F2NCl···OMe2 (Cl hole) | -869.098716 | -0.00328 | -2.058 | 3.124 | 0.005581 | 0.024647 | 0.0048 | 0.0048 |
| 26 | F3N···OEt2 | -587.381711 | -0.003822 | -2.398 | 3.173 | 0.004992 | 0.022111 | 0.0043 | 0.0043 |
| 27 | F3N···OPy345F | -974.667533 | -0.003879 | -2.434 | 3.152 | 0.004941 | 0.022464 | 0.0043 | 0.0043 |
| 28 | F3N···OPy | -677.113137 | -0.003324 | -2.086 | 3.055 | 0.006175 | 0.027526 | 0.0054 | 0.0054 |
| 29 | F2NI···NCH (I hole) | -645.206067 | -0.000866 | -0.543 | 3.344 | 0.003669 | 0.016534 | 0.0031 | 0.0031 |
| 30 | F3N···OPy345Me | -794.966152 | -0.004331 | -2.718 | 3.096 | 0.005667 | 0.024960 | 0.0049 | 0.0049 |

# 2 – Phosphorous

**Table S 2:** Summary of the used complexes for the pnictogen bond calculations. The electronic energy of the complex, the energy difference ΔE (complex – starting materials), the distance Pn···LB and the values for V(r) and G(r) are given. Values for calculation that resulted in ‘pure’ pnictogen bonding are colored in green, whereas the values that are colored in orange indicate the presence of secondary interactions (e.g. H–bonding, C···F interactions, …).

| **No.** | **Complex** | **Electronic Energy**  **[Hartree]** | **Δ E**  **[Hartree]** | **Δ E**  **[kcal/mol]** | **d (N···LB)**  **[**[**Å**](https://en.wikipedia.org/wiki/%C3%85)**]** | **V(r)**  **[a.u.]** | **ρ**  **[a.u.]** | **∇ρ^2^**  **[a.u.]** | **G(r)**  **[a.u.]** |
| --- | --- | --- | --- | --- | --- | --- | --- | --- | --- |
| 31 | F3P···NCH | -734.077732 | -0.003846 | -2.413 | 3.202 | -0.0044 | 0.008347 | 0.026834 | 0.0055 |
| 32 | F3P···NCPh | -964.953786 | -0.004498 | -2.823 | 3.153 | -0.0052 | 0.009923 | 0.030296 | 0.0064 |
| 33 | F3P···NCtBu | -891.216751 | -0.004781 | -3.000 | 3.17 | -0.005 | 0.009648 | 0.029392 | 0.0062 |
| 34 | F3P···Py | -888.816935 | -0.010065 | -6.316 | 2.662 | -0.018 | 0.029171 | 0.052262 | 0.0155 |
| 35 | F3P···NCCF3 | -1070.94305 | -0.003177 | -1.994 | 3.289 | -0.0035 | 0.006678 | 0.022725 | 0.0046 |
| 36 | F3P···NCCH3 | -773.375239 | -0.004817 | -3.023 | 3.097 | -0.0057 | 0.010463 | 0.032105 | 0.0069 |
| 37 | F3P···Py4F | -988.011117 | -0.009526 | -5.978 | 2.688 | -0.0169 | 0.027565 | 0.51568 | 0.0149 |
| 38 | F3P···Py345F | -1186.37733 | -0.008105 | -5.086 | 2.784 | -0.0133 | 0.022466 | 0.047451 | 0.0126 |
| 39 | F2PCl···NCH | -1094.33303 | -0.004131 | -2.592 | 3.167 | -0.0048 | 0.008984 | 0.028794 | 0.006 |
| 40 | F2Br···NCH | -3208.12614 | -0.004374 | -2.745 | 3.142 | -0.0051 | 0.009470 | 0.030090 | 0.0063 |
| 41 | F2PI···NCH | -931.983786 | -0.004329 | -2.716 | 3.14 | -0.0048 | 0.009553 | 0.030322 | 0.006 |
| 42 | F3P···Py4Me | -928.102676 | -0.010497 | -6.587 | 2.638 | -0.019 | 0.030603 | 0.053042 | 0.0161 |
| 43 | F3P···OMe2 | -795.641357 | -0.008078 | -5.069 | 2.778 | -0.0122 | 0.019165 | 0.052580 | 0.0127 |
| 44 | F3P···Py345Me | -1006.67081 | -0.011412 | -7.161 | 2.606 | -0.0206 | 0.032664 | 0.053702 | 0.017 |
| 45 | F3P···OPy | -963.938278 | -0.005606 | -3.518 | 2.966 | -0.0068 | 0.010401 | 0.040578 | 0.0085 |
| 46 | F3P···OPy4Me | -1003.22907 | -0.012316 | -7.728 | 2.524 | -0.0229 | 0.032131 | 0.068768 | 0.0201 |
| 47 | F3P···OPy345Me | -1081.79789 | -0.013205 | -8.286 | 2.494 | -0.0248 | 0.034250 | 0.070154 | 0.0211 |
| 48 | F3P···OPy4F | -1063.13416 | -0.011978 | -7.516 | 2.546 | -0.0216 | 0.030663 | 0.067460 | 0.0193 |
| 49 | F3P···OPy345F | -1261.49679 | -0.010274 | -6.447 | 2.648 | -0.0165 | 0.024515 | 0.061017 | 0.0159 |
| 50 | F3P···Py4I | -1185.9395 | -0.009247 | -5.803 | 2.707 | -0.0161 | 0.026506 | 0.050764 | 0.0144 |
| 51 | F3P···NCF | -833.247103 | -0.00376 | -2.359 | 3.211 | -0.0042 | 0.007900 | 0.025895 | 0.0053 |
| 52 | F3P···NCBr | -3307.33294 | -0.003972 | -2.492 | 3.188 | -0.0045 | 0.008415 | 0.027135 | 0.0056 |
| 53 | F3P···NCCl | -1193.53768 | -0.003937 | -2.471 | 3.198 | -0.0043 | 0.008206 | 0.026633 | 0.0055 |
| 54 | F3P···NCI | -1031.19945 | -0.004118 | -2.584 | 3.188 | -0.0045 | 0.008521 | 0.027349 | 0.0057 |
| 55 | F2PBr···Py345F | -3660,4263 | -0,00924 | -5,796 | 2,68 | -0,0173 | 0.028217 | 0.052964 | 0,0153 |
| 56 | F2PCl···Py345F | -1546,6329 | -0,00871 | -5,466 | 2,719 | -0,0157 | 0.025922 | 0.051335 | 0,0143 |
| 57 | F2PMe···Py | -828.852264 | -0.004754 | -2.983 | 3.027 | -0.0077 | 0.014114 | 0.036013 | 0.0083 |
| 58 | F2PH···NCH | -634.817635 | -0.002348 | -1.473 | 3.321 | -0.0033 | 0.006492 | 0.022113 | 0.0044 |
| 59 | F2PH···Py | -789.555231 | -0.00696 | -4.367 | 2.806 | -0.0129 | 0.022026 | 0.047195 | 0.0124 |
| 60 | F3P···THF | -873.007547 | -0.014975 | -9.397 | 2.734 | -0.0136 | 0.020998 | 0.055409 | 0.0137 |
| 61 | F3P···OC(CH3)2 | -833.739928 | -0.009265 | -5.814 | 2.871 | -0.009 | 0.019165 | 0.052580 | 0.0103 |

# 3 – Arsenic

**Table S 3:** Summary of the used complexes for the pnictogen bond calculations. The electronic energy of the complex, the energy difference ΔE (complex – starting materials), the distance Pn···LB and the values for V(r) and G(r) are given. Values for calculation that resulted in ‘pure’ pnictogen bonding are colored in green, whereas the values that are colored in orange indicate the presence of secondary interactions (e.g. H–bonding, C···F interactions, …). The addition of ‘Cs’ indicates that Cs symmetry was imposed for the calculation.

| **No.** | **Complex** | **Electronic Energy**  **[Hartree]** | **Δ E**  **[Hartree]** | **Δ E**  **[kcal/mol]** | **d (N···LB)**  **[**[**Å**](https://en.wikipedia.org/wiki/%C3%85)**]** | **V(r)**  **[a.u.]** | **ρ**  **[a.u.]** | **∇ρ^2^**  **[a.u.]** | **G(r)**  **[a.u.]** |
| --- | --- | --- | --- | --- | --- | --- | --- | --- | --- |
| 62 | F3As···NCH | -2628.42416 | -0.006786 | -4.258 | 3.004 | -0.0073 | 0.013051 | 0.038909 | 5.3338265 |
| 63 | F3As···Py | -2783.16708 | -0.016728 | -10.497 | 2.559 | -0.0262 | 0.038019 | 0.059918 | 12.9266854 |
| 64 | F3As···Py345Me | -2901.02026 | -0.017382 | -10.907 | 2.513 | -0.0297 | -0.041778 | 0.061455 | 14.1189525 |
| 65 | F3As···OPy | -2858.29723 | -0.021069 | -13.221 | 2.388 | -0.0381 | 0.045189 | 0.085404 | 18.6370173 |
| 66 | F3As···OPy345F | -3155.84886 | -0.018864 | -11.837 | 2.448 | -0.0321 | 0.039792 | 0.081692 | 16.4407358 |
| 67 | F3As···NCCF3 | -2965.28878 | -0.005418 | -3.400 | 3.105 | -0.0055 | 0.010127 | 0.032283 | 42.670612 |
| 68 | F3As···OPy4Me | -2897.58276 | -0.022521 | -14.132 | 2.358 | -0.0417 | 0.048250 | 0.086738 | 19.8920353 |
| 69 | F3As···OPy4F | -2957.48762 | -0.02195 | -13.774 | 2.375 | -0.0398 | 0.046603 | 0.085683 | 19.2017754 |
| 70 | F3As···OPy345Me | -2976.15202 | -0.023856 | -14.970 | 2.337 | -0.0444 | 0.050427 | 0.087679 | 20.7705479 |
| 71 | F3As···NCCl | -3087.8844 | -0.007175 | -4.502 | 3.025 | -0.0067 | 0.012277 | 0.036162 | 4.9573211 |
| 72 | F3As···NCF | -2727.59371 | -0.006878 | -4.316 | 3.048 | -0.0064 | 0.011591 | 0.034918 | 4.7063175 |
| 73 | F3As···NBr | -5201.6797 | -0.007244 | -4.546 | 2.995 | -0.0073 | 0.013102 | 0.038062 | 5.2710756 |
| 74 | F3As···OPy4NO2 | -3062.67853 | -0.016366 | -10.270 | 2.505 | -0.027 | 0.034933 | 0.077849 | 14.5582088 |
| 75 | F3As···Py345F | -3080.72535 | -0.01264 | -7.932 | 2.667 | -0.0195 | 0.030096 | 0.056224 | 10.5421512 |
| 76 | F2AsBr···Py345F | -5554,79 | -0,01331 | -8,353 | 2,632 | -0,0214 | 0.032879 | 0.057587 | 11,2324111 |
| 77 | F2AsCl···Py345F | -3441 | -0,00695 | -8,24 | 2,648 | -0,0205 | 0.031666 | 0.057117 | 10,9186566 |
| 78 | F2AsCl··NCBr | -5561,95 | -0,00695 | -4,36 | 3,09 | -0,0058 | 0.010996 | 0.032747 | 4,392563 |
| 79 | F2AsBr··NCBr | -7675,75 | -0,0069 | -4,329 | 3,019 | -0,0069 | 0.012776 | 0.036602 | 5,020072 |
| 80 | F3As···Py4F_Cs | -2882.36099 | -0.015914 | -9.986 | 2.58 | -0.0247 | 0.036288 | 0.059584 | 12.4246782 |
| 81 | F3As···NCCH3_Cs | -2667.72365 | -0.00974 | -6.112 | 3.079 | -0.0063 | 0.011746 | 0.034480 | 4.6435666 |
| 82 | F3As···Aceton_Cs | -2728.09108 | -0.016931 | -10.624 | 2.689 | -0.0162 | 0.023680 | 0.064394 | 10.1656458 |
| 83 | F3As···NCPh345Me_Cs | -2977.15309 | -0.011221 | -7.041 | 2.968 | -0.008 | 0.014277 | 0.039606 | 5.7103319 |
| 84 | F3As···Py4F | -2882.36102 | -0.015945 | -10.006 | 2.586 | -0.0243 | 0.035856 | 0.059270 | 12.2991764 |
| 85 | F3As···NCtBu | -2785.56733 | -0.011876 | -7.452 | 2.956 | -0.0083 | 0.014743 | 0.040361 | 5.7730828 |
| 86 | F3As···Py | -2783.1671 | -0.016749 | -10.510 |  | -0.0057 | 0.037643 | 0.059686 | 4.9573211 |
| 87 | F3As···Aceton | -2728.09113 | -0.016982 | -10.656 | 2.662 | -0.0174 | 0.024892 | 0.066848 | 10.7304039 |
| 88 | F3As···OMe2 | -2689.98964 | -0.01288 | -8.082 | 2.644 | -0.0189 | 0.026848 | 0.066588 | 11.1069093 |
| 89 | F3As···NCPh345Me | -2977.15543 | -0.013561 | -8.510 | 2.96 | -0.0081 | 0.014532 | 0.040091 | 5.7103319 |
| 90 | F3As···NCCH3 | -2667.72354 | -0.009636 | -6.047 | 3.076 | -0.0063 | 0.011914 | 0.034769 | 4.7063175 |
| 91 | F3As···Py4NO2 | -2987.54765 | -0.013526 | -8.488 | 2.661 | -0.0199 | 0.030701 | 0.056246 | 10.667653 |
| 92 | F3As···OPy35NO2 | -3267.04813 | -0.016608 | -10.422 | 2.539 | -0.0247 | 0.032667 | 0.074801 | 13.6169453 |

# 4 – Antimony

**Table S 4:** Summary of the used complexes for the pnictogen bond calculations. The electronic energy of the complex, the energy difference ΔE (complex – starting materials), the distance Pn···LB and the values for V(r) and G(r) are given. Values for calculation that resulted in ‘pure’ pnictogen bonding are colored in green, whereas the values that are colored in orange indicate the presence of secondary interactions (e.g. H–bonding, C···F interactions, …). The addition of ‘Cs’ indicates that Cs symmetry was imposed for the calculation.

| **No.** | **Complex** | **Electronic Energy**  **[Hartree]** | **Δ E**  **[Hartree]** | **Δ E**  **[kcal/mol]** | **d (N···LB)**  **[**[**Å**](https://en.wikipedia.org/wiki/%C3%85)**]** | **V(r)**  **[a.u.]** | **ρ**  **[a.u.]** | **∇ρ^2^**  **[a.u.]** | **G(r)**  **[a.u.]** |
| --- | --- | --- | --- | --- | --- | --- | --- | --- | --- |
| 93 | SbF3···OPy_Cs | -862.968466 | -0.017581 | -11.032 | 2.523 | -0.0317 | 0.041299 | 0.071410 | 16.8799921 |
| 94 | SbF3···OPy4Me_Cs | -902.254111 | -0.019142 | -12.012 | 2.495 | -0.0345 | 0.041420 | 0.092745 | 18.0722592 |
| 95 | SbF3···OPy4F_Cs | -962.157919 | -0.017522 | -10.995 | 2.518 | -0.0322 | 0.039508 | 0.089172 | 17.1309957 |
| 96 | SbF3···NCH_Cs | -633.101787 | -0.009688 | -6.079 | 2.943 | -0.0103 | 0.017324 | 0.045375 | 6.7770972 |
| 97 | SbF3···NCF_Cs | -732.27151 | -0.009954 | -6.246 | 2.965 | -0.0096 | 0.016303 | 0.042364 | 6.3378409 |
| 98 | SbF3···NCCl_Cs | -1092.56232 | -0.010364 | -6.504 | 2.929 | -0.0105 | 0.17744 | 0.044926 | 6.8398481 |
| 99 | SbF3···NCBr_Cs | -3206.35765 | -0.010463 | -6.566 | 2.95 | -0.0099 | 0.017055 | 0.042952 | 6.4633427 |
| 100 | SbF3···NCI_Cs | -930.224493 | -0.010949 | -6.871 | 2.955 | -0.0099 | 0.017090 | 0.042504 | 6.4633427 |
| 101 | F2SbBr···OPy_Cs | -3337.04264 | -0.017719 | -11.119 | 2.487 | -0.035 | 0.042182 | 0.092006 | 18.197761 |
| 102 | F2SbCl···OPy_Cs | -1223.24497 | -0.017467 | -10.961 | 2.502 | -0.0335 | 0.040832 | 0.090155 | 17.570252 |
| 103 | F2SbCl···OPy345Me_Cs | -1341.10065 | -0.021136 | -13.263 | 2.445 | -0.0402 | 0.046301 | 0.100133 | 20.4567934 |
| 104 | F2SbBr···OPy345Me_Cs | -3454.89845 | -0.021523 | -13.506 | 2.428 | -0.0423 | 0.047946 | 0.103082 | 21.335306 |
| 105 | F2SbBr···OPy4Me_Cs | -3376.32844 | -0.019435 | -12.196 | 2.458 | -0.0384 | 0.044925 | 0.097159 | 19.6410317 |
| 106 | F2SCl···OPy4Me_Cs | -1262.53071 | -0.019118 | -11.997 | 2.473 | -0.0367 | 0.095011 | 0.077540 | 18.9507718 |
| 107 | SbF3···NCCCH_Cs | -709.184555 | -0.009842 | -6.176 | 2.926 | -0.0108 | 0.017905 | 0.046285 | 7.0281008 |
| 108 | SbF3···NCCCCH3_Cs | -748.48208 | -0.011558 | -7.253 | 2.864 | -0.0128 | 0.020503 | 0.050825 | 7.9693643 |
| 109 | SbF3···NCCCSiH3_Cs | -999.779397 | -0.010078 | -6.324 | 2.916 | -0.0111 | 0.018318 | 0.046993 | 7.1536026 |
| 110 | SbF3···NCCCTMS_Cs | -1117.67353 | -0.011199 | -7.027 | 2.87 | -0.0126 | 0.020267 | 0.050621 | 7.9066134 |
| 111 | F2SbI···NCCCCH3_Cs symm | -946,4157 | -0,01037 | -6,507 | 2,885 | -0,0121 | 0.020607 | 0.049604 | 7,530108 |
| 112 | SbF3···NCtBu_scan | -790,24419 | -0,00117 | -7,368 | 2,95 | -0,0111 | 0.018844 | 0.047498 | 9,412635 |
| 113 | SbF3···Py_Cs | -787.847596 | -0.022513 | -14.127 | 2.582 | -0.0307 | 0.041299 | 0.071410 | 15.2484687 |
| 114 | SbF3···OPy4NO2 | -1067.3609 | -0.024006 | -15.064 | 2.47 | -0.037 | 0.042647 | 0.101376 | 19.5782808 |
| 115 | SbF3···OPy345F | -1160.532 | -0.027276 | -17.116 | 2.434 | -0.0417 | 0.046276 | 0.109440 | 21.6490605 |
| 116 | SbF3···OPy4Me | -902.266986 | -0.032017 | -20.091 | 2.372 | -0.0507 | 0.052705 | 0.125621 | 25.7906199 |
| 117 | SbF3···OPy345Me | -980.83617 | -0.033277 | -20.882 | 2.359 | -0.0528 | 0.054135 | 0.129456 | 26.7318834 |
| 118 | SbF3···NCPh | -863.982242 | -0.014741 | -9.250 | 2.873 | -0.0124 | 0.020344 | 0.047882 | 7.6556098 |
| 119 | SbF3···NCPh_Cs | -863.982747 | -0.015246 | -9.567 | 2.858 | -0.0128 | 0.020903 | 0.049155 | 7.8438625 |
| 120 | SbF3···Py4F_Cs | -887.041335 | -0.021531 | -13.511 | 2.6 | -0.0294 | 0.039943 | 0.070044 | 15.2484687 |
| 121 | SbF3···Py4I_Cs | -1084.96946 | -0.02099 | -13.171 | 2.607 | -0.0287 | 0.039306 | 0.069084 | 14.432707 |
| 122 | SbF3···Py345F_Cs | -1085.40551 | -0.018074 | -11.342 | 2.665 | -0.0241 | 0.034696 | 0.063169 | 12.55018 |
| 123 | SbF3···OPy4NO2_Cs | -1067.3609 | -0.024006 | -15.064 | 2.47 | -0.037 | 0.042647 | 0.101376 | 19.5782808 |
| 124 | SbF3···OPy35NMe2_Cs | -1130.71263 | -0.035274 | -22.135 | 2.343 | -0.0554 | 0.055784 | 0.134509 | 27.9241505 |

# 5 – Bismuth

**Table S 5:** Summary of the used complexes for the pnictogen bond calculations. The electronic energy of the complex, the energy difference ΔE (complex – starting materials), the distance Pn···LB and the values for V(r) and G(r) are given. Values for calculation that resulted in ‘pure’ pnictogen bonding are colored in green, whereas the values that are colored in orange indicate the presence of secondary interactions (e.g. H–bonding, C···F interactions, …). The addition of ‘Cs’ indicates that Cs symmetry was imposed for the calculation.

| **No.** | **Complex** | **Electronic Energy**  **[Hartree]** | **Δ E**  **[Hartree]** | **Δ E**  **[kcal/mol]** | **d (N···LB)**  **[**[**Å**](https://en.wikipedia.org/wiki/%C3%85)**]** | **V(r)**  **[a.u.]** | **ρ**  **[a.u.]** | **∇ρ^2^**  **[a.u.]** | **G(r)**  **[a.u.]** |
| --- | --- | --- | --- | --- | --- | --- | --- | --- | --- |
| 125 | BiF3···OPy_Cs | -837.332385 | -0.018323 | -11.498 | 2.664 | -0.0249 | 0.030707 | 0.094428 | 15.2484687 |
| 126 | BiF3···OPy4Me_Cs | -876.617899 | -0.019753 | -12.395 | 2.64 | -0.0272 | 0.032897 | 0.098470 | 16.2524831 |
| 127 | BiF3···OPy4F_Cs | -936.521626 | -0.018052 | -11.328 | 2.662 | -0.0244 | 0.031008 | 0.094677 | 15.8132268 |
| 128 | BiF3···NCH_Cs | -607.467431 | -0.012155 | -7.627 | 2.96 | -0.0113 | 0.052553 | 0.043694 | 7.5928589 |
| 129 | BiF3···NCF_Cs | -706.637642 | -0.012909 | -8.101 | 2.957 | -0.0096 | 0.017748 | 0.052037 | 6.3378409 |
| 130 | BiF3···NCCl_Cs | -1066.92846 | -0.013329 | -8.364 | 2.937 | -0.0118 | 0.018580 | 0.053266 | 7.9066134 |
| 131 | BiF3···NCBr_Cs | -3180.72391 | -0.013546 | -8.500 | 2.95 | -0.0116 | 0.018363 | 0.052508 | 7.7811116 |
| 132 | BiF3···NCI_Cs | -904.590971 | -0.01425 | -8.942 | 2.948 | -0.0116 | 0.018440 | 0.052219 | 7.7183607 |
| 133 | F2BiBr···OPy_Cs | -3311.41548 | -0.018411 | -11.553 | 2.619 | -0.0285 | 0.034542 | 0.099680 | 16.7544903 |
| 134 | F2BiCl···OPy_Cs | -1197.61615 | -0.018344 | -11.511 | 2.633 | -0.0273 | 0.033268 | 0.097985 | 16.2524831 |
| 135 | F2BiCl···OPy345Me_Cs | -1315.4716 | -0.021786 | -13.671 | 2.579 | -0.0322 | 0.038117 | 0.108256 | 18.7625191 |
| 136 | F2BiBr···OPy345Me_Cs | -3429.27105 | -0.021978 | -13.791 | 2.565 | -0.0341 | 0.039429 | 0.110336 | 19.3272772 |
| 137 | F2BiBr···OPy4Me_Cs | -3350.70114 | -0.019988 | -12.543 | 2.593 | -0.031 | 0.036721 | 0.104931 | 17.9467574 |
| 138 | F2BiCl···OPy4Me_Cs | -1236.90179 | -0.019901 | -12.488 | 2.608 | -0.0297 | 0.035492 | 0.102555 | 17.3819993 |
| 139 | BiF3···NCCCH_Cs | -683.550288 | -0.012398 | -7.780 | 2.931 | -0.0121 | 0.0189530 | 0.055445 | 8.157617 |

Figure S 1: QTAIM plots for the PnF_3_∙∙∙Pyridine complexes. Red rectangles indicate the formation of secondary non-covalent interactions. The pnictogen-bond energies were calculated using the derived predictors and are given in kcal/mol.
